# Results of a prospective observational study of autologous peripheral blood mononuclear cell therapy for no-option critical limb-threatening ischemia and severe diabetic foot ulcers

**DOI:** 10.1186/s12933-022-01629-y

**Published:** 2022-09-28

**Authors:** Andrea Panunzi, Fabiana Madotto, Elena Sangalli, Federica Riccio, Adriana Barbara Sganzaroli, Paolo Galenda, Amelia Bertulessi, Maria Francesca Barmina, Ornella Ludovico, Orazio Fortunato, Francesco Setacci, Flavio Airoldi, Davide Tavano, Laura Giurato, Marco Meloni, Luigi Uccioli, Antonino Bruno, Gaia Spinetti, Carlo Maria Ferdinando Caravaggi

**Affiliations:** 1 Diabetic Foot Dpt, IRCCS MultiMedica, Milan, Italy; 2 Fondazione IRCCS Istituto Nazionale dei Tumori, Milan, Italy; 3 Diabetic Foot Unit, University of Rome Tor Vergata, Rome, Italy; 4 Laboratory of Immunology and General Pathology, Department of Biotechnologies and Life Sciences, University of Insubria, Varese, Italy; 5 Laboratory of Cardiovascular Pathophysiology-Regenerative Medicine, IRCCS MultiMedica, Milan, Italy; 6 CTO Andrea Alesini Hospital, Division of Endocrinology and Diabetes, Department of Systems Medicine, University of Rome Tor Vergata, Rome, Italy; 7 Laboratory of Innate Immunity, Unit of Molecular Pathology, Biochemistry and Immunology, IRCCS MultiMedica, Milan, Italy; 8 Value-based Healthcare Unit, IRCCS MultiMedica, Milan, Italy; 9 Interventional Cardiology Unit, IRCCS MultiMedica, Milan, Italy; 10 Vascular Surgery Unit, IRCCS MultiMedica, Milan, Italy; 11 Unit of Endocrinology, IRCCS Casa Sollievo della Sofferenza Hospital, San Giovanni Rotondo, Italy

**Keywords:** Critical limb-threatening ischemia, Diabetes mellitus, Autologous cell therapy

## Abstract

**Background:**

Cell therapy with autologous peripheral blood mononuclear cells (PB-MNCs) may help restore limb perfusion in patients with diabetes mellitus and critical limb-threatening ischemia (CLTI) deemed not eligible for revascularization procedures and consequently at risk for major amputation (no-option). Fundamental is to establish its clinical value and to identify candidates with a greater benefit over time. Assessing the frequency of PB circulating angiogenic cells and extracellular vesicles (EVs) may help in guiding candidate selection.

**Methods:**

We conducted a prospective, non-controlled, observational study on no-option CLTI diabetic patients that underwent intramuscular PB-MNCs therapy, which consisted of more cell treatments repeated a maximum of three times. The primary endpoint was amputation rate at 1 year following the first treatment with PB-MNCs. We evaluated ulcer healing, walking capability, and mortality during the follow-up period. We assessed angiogenic cells and EVs at baseline and after each cell treatment, according to primary outcome and tissue perfusion at the last treatment [measured as transcutaneous oxygen pressure (TcPO_2_)].

**Results:**

50 patients were consecutively enrolled and the primary endpoint was 16%. TcPO_2_ increased after PB-MNCs therapy (17.2 ± 11.6 vs 39.1 ± 21.8 mmHg, p < .0001), and ulcers healed with back-to-walk were observed in 60% of the study population (88% of survivors) during follow-up (median 1.5 years). Patients with a high level of TcPO_2_ (≥ 40 mmHg) after the last treatment showed a high frequency of small EVs at enrollment.

**Conclusions:**

In no-option CLTI diabetic patients, PB-MNCs therapy led to an improvement in tissue perfusion, a high rate of healing, and back-to-walk. Coupling circulating cellular markers of angiogenesis could help in the identification of patients with a better clinical benefit over time.

**Supplementary Information:**

The online version contains supplementary material available at 10.1186/s12933-022-01629-y.

## Background

Critical limb-threatening ischemia (CLTI), the most advanced form of peripheral arterial disease (PAD) [1–3], is an independent risk factor for major amputation in diabetic patients. Nowadays a great number of patients affected by CLTI belong to the diabetes population, accounting for about 10.5% of the US citizens and presenting with a specific diabetic arterial disease [4]. Due to the large involvement of Below-the-Knee (BTK) and Below-the-Ankle (BTA) arteries in diabetic PAD, the peripheral endoluminal approach by Percutaneous Transluminal Angioplasty (PTA) has become the first choice, even in the presence of diabetes-associated comorbidities, such as cardiovascular disease, renal impairment, and hemodialysis [4]. Despite the high success rate of PTA, clinical restenosis still represents a huge problem, reaching around 70% at 1-year follow-up [5, 6]. In addition, although early revascularisation procedures lead to a high rate of limb salvage [7], several PTA fails due to very distal BTK disease with involvement of the tibial-peroneal-trunk, anterior and posterior tibial arteries, peroneal artery, and ultra BTA disease, with involvement of pedal and plantar arteries [8, 9]. These obstructive patterns are considered as no-option CLTI and they account for approximately 25% of patients [8, 10]. No-option CLTI represents a predictor of non-healing ulcers, failure of surgical approaches with a high risk of major amputation, and mortality [10]. All of the above provide the rationale for the use of advanced therapies for no-option patients, such as implanting autologous MNCs, that can be isolated from the bone marrow (BM) or the PB [11, 12]. This procedure is safe and effective, as indicated by a series of randomized and not randomized clinical trials [13]. However, the high heterogeneity in terms of the cell types and cell subsets within the cell preparation, method of cell injection, and the lack of a clear definition of the associated cellular mechanisms call for new studies. Moreover, assessing the frequency of circulating markers of angiogenesis could guide candidate selection to improve the therapy’s benefits over time.

Here, we report the results of a prospective non-controlled observational study on no-option CLTI subjects with diabetes and ischemic foot ulcers that received intramuscular injections of PB-MNCs. In addition to the major amputation rate, we evaluated ulcer healing, walking capability, and mortality during the whole follow-up period. Moreover, we evaluated cellular indicators of angiogenesis (at enrollment and during cell therapy) to identify subgroups of patients who may have a greater benefit from therapy. In particular, we studied the frequency of classical pro-angiogenic hematopoietic stem and progenitor cells (HSPCs) (CD34^+^ and CD34^+^CXCR4^+^) and small-size cell-derived extracellular vesicles (EVs), referred to as exosomes that showed association with angiogenic capabilities in preclinical studies [14, 15].

## Methods

### Study design

We conducted a prospective observational non-controlled study on a cohort of consecutively enrolled diabetic patients affected by CLTI with a low level of transcutaneous oxygen pressure (TcPO_2_ < 30 mmHg) and ulcer with Texas University Class (TUC) 3D lesion not suitable for revascularization procedures (neither surgical nor endoluminal) or with insufficient improvement in blood flow after bypass or PTA (no-option) (see the Enrollment section for a detailed description).

*Inclusion criteria*: (1) age 18 years and older; (2) CLTI as pain at rest and/or not infected ulcer or dry gangrene due to arteriopathy, TcPO_2_ < 30 mmHg or ankle pressure > 70 mmHg (TASC criteria); (3) patient not eligible for surgery or endovascular procedure (non-passable obstruction); (4) patient underwent a procedure without a clinical recovery (value of TcPO_2_ after PTA remains < 30 mmHg).

*Exclusion criteria*: (1) neoplastic diseases; (2) recent acute myocardial infarction or ictus; (3) clinical conditions that determine life expectancy less than 6 months (a severe cardiovascular disease where life expectancy is investigated using NYHA classification or cancer disease when a clinical condition like catabolic clinical aspect or sarcopenia drive the decision); (4) dialysis for chronic kidney disease; (5) ischemic lesions that require immediate amputation or risk of death; (6) extensive necrosis of the limb or other conditions that require amputation; (7) clinically active infection with antibiotic therapy; (8) pregnancy or breastfeeding; (9) drug or alcohol abuse; (10) lack of signed informed consent.

All patients underwent cell therapy with a concentrated solution of autologous PB-MNCs, produced by point of care HemaTrate^®^ Blood Filtration System (Cook Regentec –Indianapolis, Indiana–USA). Participants were treated by injecting a concentrate of MNCs obtained from 120 ml of PB (Additional file 1: Fig. S1) [16]. PM-MNCs therapy could be repeated a maximum of three times (treatments) every 45 days, according to clinical judgment.

**Fig. 1:**
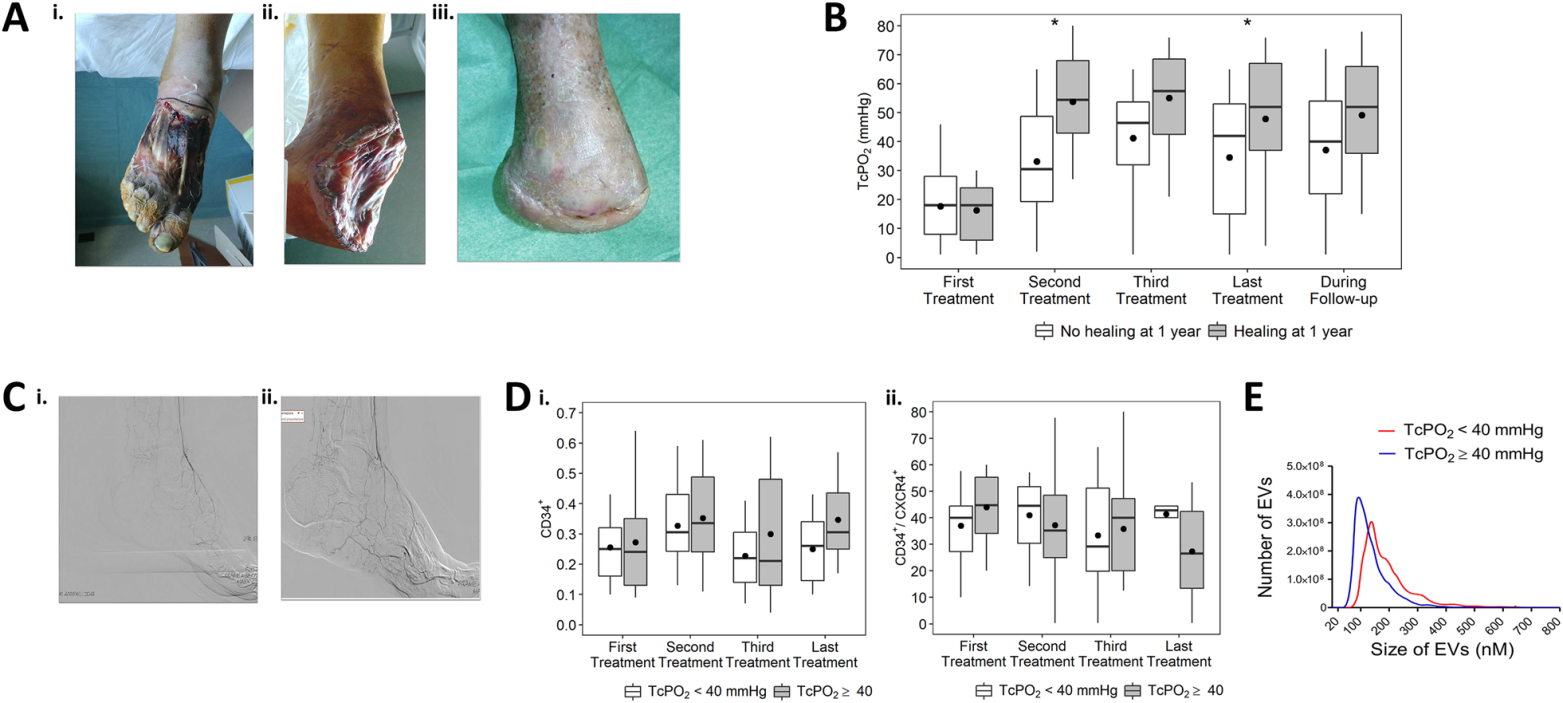
Panel A. Representative pictures of a clinical case of PB-MNCs therapy and right Chopart Amputation. From left to right: (i) before therapy; (ii) at the time of the 1st intramuscular PB-MNCs treatment; (iii) ulcer healing. Panel B. TcPO_2_ values in study population stratified by healing without a major amputation after 1 year. The grey box represents patients with healing and the white box represents patients without healing at 1-year follow-up. *Comparison between groups, p-value < 0.05. Panel C. Representative angiograms of two no-option CLTI diabetic patients. From left to right: (i) patient after unsuccessfully PTA (TcPO_2_ < 30 mmHg) before PB-MNCs therapy showing the typical “desert” foot condition; (ii) patient after 2 months from PB-MNCs therapy showing collateral vascular remodeling. Panel D. Relative abundance of CD34^+^ and CD34^+^CXCR4^+^ cell subpopulations during PB-MNCs therapy stratified by TcPO_2_ values observed after the last PB-MNCs treatment (< 40 mmHg; ≥ 40 mmHg). The grey box represents patients with high levels of TcPO_2_ (≥ 40 mmHg) and the white box represents patients with low levels (< 40 mmHg). Panel E. Extracellular vesicles size distribution according to TcPO_2_ levels after the last PB-MNCs treatment (< 40 mmHg; ≥ 40 mmHg)

The study included follow-up visits twice a month for 12 months from enrollment (day of first treatment) with measurement of TcPO_2_ levels and motor ability (back-to-walk). Information on the vital status and execution of major amputation was recorded 1 year from enrollment and until the end of follow-up (March 3rd, 2021).

At enrollment and after each treatment, we assessed the frequency of markers of angiogenesis (pro-angiogenic HSPCs (CD34^+^ and CD34^+^CXCR4^+^) measured in the PB by multicolor flow cytometry) and levels of isolated EVs evaluated by Nanoparticle Tracking Analysis as detailed below.

The primary endpoint was the major amputation rate observed 1 year from enrollment and we also evaluated ulcer healing, walking capability, and mortality during the follow-up period.

The study protocol complied with the principles stated in the Declaration of Helsinki and was approved by the Ethical Committee of IRCCS MultiMedica (protocol number 332.2018).

### Enrollment

Participants were consecutively enrolled from August 2018 to July 2019, during a clinical outpatient visit in the Diabetic Foot Department of IRCCS MultiMedica consisting of a vascular assessment (pedal pulses and TcPO_2_) and evaluation and classification of foot ulcers. Subjects meeting all inclusion study criteria and who did not meet any exclusion criteria were enrolled. Each participant was subjected to a blood test, chest and foot X-ray, angiogram, and percutaneous transluminal angioplasty (PTA) with artery color doppler for controlling vascular accesses. Patients who were not suitable for PTA (non-passable obstruction) or value of TcPO_2_ after PTA remains < 30 mmHg were consecutively enrolled and admitted the next day to the operating room for autologous PB-MNCs therapy and surgical treatment of the foot ulcer.

### Description of therapeutic CLTI treatment procedures

#### PB-MNCs concentration

PB-MNCs concentration was produced by the point of care HemaTrate^®^ Blood Filtration System (Cook Regentec—Indianapolis, Indiana—USA). The cell product obtained by gravity filtration has been previously extensively characterized in terms of composition, recovery, and cytofluorimetric cell population analysis [16]. One hundred and twenty ml of acid-citrate-dextrose (ACD)-anticoagulated peripheral blood was loaded in the upper blood bag and gravity filtration was allowed. The captured MNCs were harvested by sterile saline backflush and immediately implanted. All the procedures were performed in the operatory/surgery room with anaesthesiologic support (propofol and/or peripheral block).

#### Cells implants and surgical procedure description

After appropriate surgical debridement of the wound bed, multiple perilesional and intramuscular injections of PB-MNCs suspension (0.2–0.3 cc in boluses) were injected along the relevant axis below the knee, at intervals of 1–2 cm and to a mean depth of 1.5–2 cm, using a 21G needle. Representative pictures of a clinical case are shown in Additional file 1: Fig. S1. Since patients may present different occlusive patterns, the choice of suitable leg and foot area for intramuscular injection represents a key factor for the success of the procedure. Considering paracrine activity of PB-MNCs subpopulations on angiogenesis we avoided treatment of vessels with very long obstructive disease. The treatment choice was addressed to vessels with short segmental obstruction where few collaterals were visible by angiography. The dorsal and plantar aspects of the foot were always treated as peri-wound areas. During the first treatment, surgeons performed only minimal surgery (removal of infection and necrotic tissue), followed by dressing with Hyaluronic Acid (HA) dermal substitute with grease gauze as a secondary dressing. In subsequent treatments, if possible and necessary based on clinical judgment, surgeons proceeded on with surgical debridement and HA dermal substitute and grease gauze in case of healing by second intention or definitive surgical procedure when possible (toe, ray amputation, or midfoot amputation). As soon as possible, patients were allowed to walk with industrial postoperative shoes with offloading insole, to avoid hypokinetic bed rest syndrome very dangerous for aged patients.

### Peripheral blood analyses

#### Characterization of MNC subpopulations by multicolor flow cytometry

Total PB-MNCs were isolated from peripheral blood (4 mL of whole blood, in ethylene di-amine tetra-acetic acid (EDTA) anticoagulant tubes), by stratification on Ficoll histopaque. Plasma was stored for EV purification [14]. MNCs were phenotypically characterized by multicolor flow cytometry, using a BD FACS Canto II analyzer. Briefly, 1X10^5^ cells/FACS tube were stained in 100 µL of PBS for 30 min at Room Temperature (RT) in the dark, with the following monoclonal anti-human antibodies: FITC-conjugated CD45 (REA747), PE-conjugated CXCR4 (12G5), and PE-Cy7-conjugated CD34 (581), from BD Biosciences. Following staining, cells were washed in PBS at 1200xg for 5 min, RT. Samples were immediately acquired. Viable cells were gated based on FSC-A/SSC-A morphology, then interrogated for specific surface antigen expression. Cell populations were identified as follows: CD45^dim^CXCR4^+^CD34^+^ cells (CD34^+^ cells) (Additional file 1: Fig. S2).

#### Extracellular vesicles (EVs) isolation and analysis

One-hundred μL of plasma was used for the EV isolation with a ready-made chromatography method known to eliminate > 95% of non-vesicular proteins (Exo-spin Blood, Cell Guidance Systems, Cambridge, UK) [17, 18]. EV purity and quantity were measured by Nanoparticle Tracking Analysis, using Nanosight NS300 (Malvern Panalytical Ltd) [19].

#### Statistical analysis

Since longitudinal data on the recovery for non-option diabetic patients was almost scarce in the literature when planning the study, the sample size was determined according to the percentage of avoided major amputation after 1 year from treatment, assuming that 70% of avoided amputations represented a clinical success factor. We estimated that a sample size of 44 patients was necessary to reach a power of 85% and a type I error of 5% (one-side). We decided to enroll consecutively 50 patients, considering a drop-out rate of around 5%.

Descriptive statistics included proportions for categorical and mean (standard deviation (SD)) or median (interquartile range) for continuous variables, according to the skewness of data distribution. No assumptions were made for missing data.

The study population was stratified according to the ulcer healing after 1 year from treatment without a major amputation and statistical differences in proportions between groups were assessed with the chi-square test, or Fisher exact test according to the number of expected cases. Continuous variables were compared using the T-test or Wilcoxon rank-sum test, according to Normal data distribution. Shapiro-Wilks test was used to assess normality in data distribution. The same approaches were used when the study population was stratified according to the TcPO_2_ level observed during the last treatment of PB-MNCs therapy (< 40 mmHg; ≥ 40 mmHg).

Kaplan–Meier approach was applied to assess the probability of healing without amputation according to the TcPO_2_ level at the last treatment of PB-MNCs therapy (< 40; ≥ 40 mmHg).

All p-values were two-sided, with p-values < 0.05 considered statistically significant. Statistical analyses were performed with R, version 3.5.2. (R Project for Statistical Computing, http://www.R-project.org) and SAS software, version 9.4 (SAS Institute, Cary, NC, USA).

## Results

### Cell therapy with autologous PB-MNCs in no-option CLTI diabetic patients results in a high rate of recovery

In the period from August 2018 to July 2019, we consecutively enrolled 50 no-option CLTI diabetic patients (Table 1). The mean age was 75 years (SD 10) and 62% were males. Ulcers were all more than 5 cm^2^ presenting with gangrene. After foot surgical procedure infection was present in 40% of patients and osteomyelitis in 60%. The vessels affected were 80% anterior and posterior tibial arteries.

**Table 1:** Demographic, clinical, and ulcer characteristics after foot surgical procedure

	Study population
N	50
Males, n (%)	31 (62.0)
Age (years), mean ± SD	75.2 ± 10.0
Type 2 diabetes mellitus, n (%)	50 (100.0)
Duration of diabetes (years), mean ± SD	21.0 ± 8.6
Ischemic heart disease, n (%)	29 (58.0)
PB-MNCs treatments, n (%)	
1	12 (24.0)
2	10 (20.0)
3	28 (56.0)
Ulcer characteristic	
Right limb, n (%)	23 (46.0)
Dimension > 5 cm^2^, n (%)	50 (100.0)
Infection, n (%)	20 (40.0)
Gangrene, n (%)	50 (100.0)
Osteomyelitis, n (%)	30 (60.0)
Heel location, n (%)	7 (14.0)
Distribution of arterial lesions and vascular findings	
Vessels affected, mean ± SD	3.8 ± 1.8
Iliac arteries, n (%)	1 (2.0)
CFA, n (%)	3 (6.0)
Profunda artery, n (%)	2 (4.0)
SFA, n (%)	28 (56.0)
Popliteal artery, n (%)	20 (40.0)
TPT, n (%)	8 (16.0)
ATA, n (%)	40 (80.0)
Peroneal artery, n (%)	22 (44.0)
PTA, n (%)	40 (80.0)
Pedidialartery, n (%)	12 (24.0)
Plantar arteries, n (%)	12 (24.0)

*ATA* anterior tibial artery, *CFA* common femoral artery, *PB-MNCs* peripheral blood mononuclear cells, *PTA* posterior tibial artery, *SD* standard deviation, *SFA* superficial femoral artery, *TPT* tibiperoneal trunk

After 1 year from enrollment, 8 (16%) patients underwent major amputation and the number reached 9 (18%) at the end of the follow-up period (median time 1.5 years) (Table 2). Due to the obstruction pattern of leg arteries, all patients were at high risk for Above-the-Knee (ATK) amputation (data not shown). Of note, of the total 9 major amputations, 5 were BTK and 4 were ATK. After 1 year, 16 patients (32%) died, and 47% of surviving patients had healed without a major amputation with an estimated mean healing time of 257 days. At the end of follow-up, 88.5% of the alive patients (n = 26) showed a good quality of residual walking by wearing industrial or custom-made shoes and sandals with a molded insole. Patients submitted to BTK amputation returned to walk with a transtibial orthosis (Fig. 1 panel A, Additional file 1: Fig. S3). In addition to limb salvage, since the return to good residual walking capacity should be considered the key point of care for this class of patients, we considered the rate of patients that returned to walking. In our cohort, the ulcer healed with back-to-walk in 30 on 50 patients (60%).

**Table 2:** Clinical outcomes occurred during follow-up

	Study population (n = 50)
At 1 year	
Dead, n (%)	16 (32.0)
Major amputation, n (%)	
All patients (n = 50)	8 (16.0)
Alive at 1 year (n = 34)	7 (20.6)
Ulcer healing, n (%)	
All patients (n = 50)	22 (44.0)
Alive at 1 year (n = 34)	21 (61.8)
Ulcer healing without a major amputation, n (%)	
All patients (n = 50)	17 (34.0)
Alive at 1 year (n = 34)	16 (47.1)
Estimated* time to healing without a major amputation (days), mean ± SE	257 ± 17
At the end of follow-up	
Follow-up time (years), median [IQR]	1.46 [0.55–2.01]
Dead, n (%)	24 (48.0)
Major amputation, n (%)	
All patients (n = 50)	9 (18.0)
Alive at the end of follow-up (n = 26)	5 (19.3)
Walking capability, n (%)	
All patients (n = 50)	30 (60.0)
Alive at the end of follow-up (n = 26)	23 (88.5)
Ulcer healing, n (%)	
All patients (n = 50)	30 (60.0)
Alive at the end of follow-up (n = 26)	23 (88.5)
Ulcer healing without a major amputation, n (%)	
All patients (n = 50)	25 (50.0)
Alive at the end of follow-up (n = 26)	19 (73.1)
Estimated^*^ time to healing without a major amputation (days), mean ± SE	345 ± 39

*IQR* interquartile range [1st quartile-3rd quartile], *SE* standard error

^*^Time was estimated with Kaplan–Meier approach, considering deaths and drop-out as censored

### Healing is associated with tissue oxygenation

Next, we investigated whether the healing without major amputation was associated with tissue perfusion, described as an increase in TcPO_2_ levels. Mean TcPO_2_ at enrollment was 17.2 mmHg (SD 11.5) and it statistically increased up to 39.1 mmHg (SD 21.7) (p < 0.0001), as shown in Additional file 1: Table S1. Patients who recovered without a major amputation after 1 year showed higher TcPO_2_ levels at the end of PB-MNCs therapy (47.9 ± 22.1) compare to those without ulcer healing (34.6 ± 20.5) (p = 0.0395) (Fig. 1B and Additional file 1: Table S1). At the end of the follow-up, TcPO_2_ values remained stable in both groups (49.2 ± 20.2 vs 39.3 ± 22.8, p = 0.1265) (Fig. 1B and Additional file 1: Table S1). The probability of healing without a major amputation during follow-up was higher in subjects with TcPO_2_ > 40 mmHg after the last cell treatment, but it did not statistically differ from that observed in patients with low TcPO_2_ levels (Additional file 1: Fig. S4). Four post-treatment angiographies were performed in patients deemed eligible for the procedure (with glomerular filtration rate > 30 ml/min) and showed an increase in collateral vessels indicating vascular remodeling (Fig. 1C).

### Characterization of the angiogenic cells and extracellular vesicles pattern in PB

Using multicolor flow cytometry (Additional file 1: Fig. S2), we next assessed the frequency in the PB of classical angiogenic MNCs (namely HSPCs of the CD34^+^ and CD34^+^CXCR4^+^ classes).

Stratifying the studied cohort according to healing without major amputation, we did not observe statistical differences in the relative cell frequency of any of the two angiogenic subpopulations studied (Additional file 1: Table S1, and Additional file 1: Fig. S5). To further investigate the relationship between angiogenic circulating cells and increased microvascular density responsible for perfusion, we studied the association of cell frequency with tissue oxygenation at the end of PB-MNCs therapy, stratifying the population into two groups (TcPO_2_ < 40 and ≥ 40 mmHg) (Additional file 1: S2, Table 3). It was confirmed that TcPO_2_ improvement was more remarkable in cases where PB-MNCs therapy could be performed three times. No significant association was observed between CD34^+^ and CD34^+^CXCR4^+^ frequency and TcPO_2_ (Fig. 1D). Since the angiogenic activity of circulating CD34^+^ cells is at least in part associated with EVs transfer of signals to the endothelium [14, 15], we measured the concentration and the percentage of small-size EVs (30-100 nM of diameter), usually referred to as exosomes. Data show that the group with high levels of TcPO_2_ (≥ 40 mmHg) was characterized by smaller size EVs and a higher concentration of exosomes (30-100 nM EVs) in the plasma with respect to the group with TcPO_2_ ≤ 40 mmHg (Fig. 1E and Table 3).

**Table 3:** Cellular and molecular characterization at enrollment, stratifying study population according to TcPO_2_ levels after the last PB-MNCs treatment (< 40 mmHg; ≥ 40 mmHg)

	TcPO_2_ levels		Comparison, p-value
	< 40 mmHg	≥ 40 mmHg	
N (%)	21 (42.0)	29 (58.0)	–
Age (years), mean ± SD	78.67 ± 8.78	72.69 ± 10.24	0.0522
Males, n (%)	12 (57.14)	19 (65.52)	0.5471
PB-MNCs treatments, n (%)			
1	12 (57.14)	0 (0.00)	** <0 .0001**
2	1 (4.76)	10 (34.48)	
3	8 (38.10)	19 (65.52)	
TcPO_2_, mean ± SD			
At enrollment	16.48 ± 11.54	17.72 ± 11.74	0.5588
After the last PB-MNCs treatment	17.10 ± 12.20	55.07 ± 9.73	** <0 .0001**
PB cell frequency^*^, mean ± SD			
CD34^+^	0.26 ± 0.12	0.30 ± 0.21	0.7215
CD34^+^CXCR4^+^	40.56 ± 19.78	44.06 ± 11.48	0.2880
EVs^‡^, mean ± SD			
EV size (nm)	195.59 ± 29.74	136.31 ± 27.41	**0.0001**
Total EV concentration (ml × 10^10^)	3.50 ± 1.20	3.62 ± 1.72	0.9728
Small EV (30-100 nM) (%)	4.0 ± 5.0	33.0 ± 18.0	**0.0012**

*EVs* extracellular vesicles, *PB* peripheral blood, *PB-MNCs* peripheral blood mononuclear cells, *SD* standard deviation

^*^CD34^+^ was not available for 5 patients (10%) s, CD34^+^CXCR4^+^ for 10 patients (20%). ‡ EV (30-100 nM) evaluation was performed on 22 patients (44%)

P-values < 0.05 are displayed in bold

## Discussion

CLTI, the more advanced form of PAD, represents a life-threatening condition for a high proportion of patients with diabetes. Autologous cell therapy with PB-MNCs is an approach that promises to change the current care. We here presented the results of an observational study in long-term followed-up no-option CLTI diabetic subjects. Of note, we showed that autologous cell therapy results in a high rate of ulcer healing with back-to-walk a crucial clinical aspect too often neglected in the care of this class of patients [20]. To our knowledge, this is the first study where “limb salvage” was defined as a patient obtaining “limb salvage” only together with back-to-walk with a good residual walking ability for a long period of follow-up.

The mortality rate at 1 year in our cohort was in line with the international literature for the population enrolled, thus we exclude that mortality could be due to the PB-MNC treatment. E.g. Jones et al. reported that among PAD 186,338 patients who underwent major lower limb amputation, the mortality rate was 13.5% at 30 days, 48.3% at 1 year, and 70.9% at 3 years [21]. Another study on 574 no-option CLI patients (of which 70% were diabetic), reported a 23% major amputation rate and a 31.6% death rate, primarily from cardiovascular disease, after 2 years. Around 25% of diabetic CLTI patients are no-option CLTI and may not be treated either by surgical or endoluminal approaches [23]. In the last 10 years, different approaches have been proposed such as veins arterialization, Hybrid Foot Vein arterialization, or Percutaneous deep venous arterialization, with promising results on limb salvage and ulcer healing rate [24–26]. However, the small number of treated patients, mixed studied population, and lack of information about the number of patients who started walking again curb the enthusiasm for those approaches. Moreover, fragile no-option CLTI patients are often non-suitable for bypass or very complicated endoluminal procedures.

Thus, obtaining 60% of limb salvage with about 100% of back-to-walk patients affected by CLTI no-option with foot infections after surgical treatment or gangrene with a feasible procedure, such as injection of not selected autologous PB-MNCs as we observed here, may represent a valid clinical approach in CLTI management and patients’ quality of life. Several patients in this study felt a dramatic reduction of pain after PB-MNCs treatment with a reduction of the need for drugs like morphine; going back to walking with postoperative protective shoes resulted in an improvement in quality of life and reduction of hypokinetic bed rest syndrome. These aspects deserve a dedicated study for confirmation.

Successful PTA performed in BTK and BTA areas pay a risk of clinical restenosis around 70% at 1-year follow-up. In this study, autologous cell therapy by multiple peri-lesional and intramuscular injections of PB-MNCs suspension resulted in a steady increase in the TcPO_2_ levels that reached the maximum level after the 2nd treatment with a long period of maintenance of high TcPO_2_ values. This data together with the absence of signs of ischemia at the level of the feet indicate the importance of this cell therapy. Autologous cell therapy potentially activates collateral vessel growth, resulting in vessel remodeling, as we highlighted by post-treatment angiograms. The blood perfusion of soft tissue and muscles could explain the high rate of patients healed and low risk of clinical restenosis in a long period of follow-up.

The molecular mechanisms of new vessel growth may be related to the ability of the injected PB-MNCs to induce vascular remodeling, by paracrine activity (delivering growth factors, cytokines, angiogenic coding, and non-coding RNAs, either free or encapsulated in EVs of small size-exosomes, to soft tissue), as has been suggested by the results in preclinical studies [27–29]. Of note, in line with this hypothesis, we here observed that patients with higher tissue perfusion at the end of the follow-up were characterized by a higher number of small-size EVs.

Lastly in this study classic cellular markers of the angiogenic response to therapy were assessed. The frequency of CD34^+^ angiogenic cells did not show an association with the treatment. This result deserves further investigation, especially considering our previous data showing CD34^+^HSC functional impairment in subjects with diabetic CLTI centered on the miR-21 molecular pathway [14, 30, 31]. The low number of cases did not allow us for further cellular analyses.

Moreover, other MNC subpopulations may be implicated in angiogenesis, e.g. monocyte subsets (classical, non-classical, intermediate), T cell subsets, and B cells, and new studies are needed to depict the complexity of leukocyte-vascular cell interactions in this clinical setting.

A major limitation of this study resides in its not-controlled nature. We are aware that a randomized controlled trial (RCT) would have allowed gaining evidence in support of indications for PB-MNCs in the treatment of no-options CLTI. However, considering the high risk of major amputations in the cohort of no-option CLTI, as described in many observational studies [32, 33], it was not ethical in our opinion to perform an RCT leaving patients non-suitable for by-pass o successful PTA, without any treatment. Of note, this was the case also for the inclusion of PTA in the guidelines for patients not suitable for by-pass due to comorbidities or insufficient run-off due to vascular occluded pattern. Although no RCT was done before, many prospective and retrospective observational studies demonstrated that PTA was safe and effective in the treatment of diabetic arterial disease.

## Conclusions

We here provide new evidence in support of autologous PB-MNC therapy in no-option diabetic CLTI. PB-MNC treatment as adjuvant therapy in diabetic patients was proposed by Persiani et al. [34] and discussed in a recent review on autologous cell therapy [35]. PB-MNCs treatment may find application in nondiabetic contexts. Further studies in larger cohorts are needed to confirm these data and to test this treatment for other high-risk patients (e.g. those on hemodialysis and for the ulcers of many autoimmune diseases). However, the results presented here provide a successful example of real-life CLTI patient management and are of great clinical importance since limbs salvage, rapid return to walk with good residual walking capability, reduction in mortality and an improvement in quality of life are the main objective of diabetic foot care.

## Supplementary Information

**Additional file 1**: **Figure S1** Panel A-B. Representative case of gangrene of the big toe. Panel C. PB-MNCs injection procedure was performed along the relevant axis below the knee, below the ankle, and closed to the wound area, at intervals of 1–2 cm and to a mean depth of 1.5–2 cm, using a 21G needle.Panel D. Patient submitted to big toe necrosectomy with bone exposure and dressing with Bioinductive Dermal Substitute based on Hyaluronic Acid. **Figure S2** Representative dot plots for the gating strategy to detect the relative frequency (expressed as % of cells positive for the selected surface antigens and their combination) of the following PB-MNCs populations, as determined by multicolor flow cytometry. For rare events (CD34^+^ and CD34^+^CXCR4^+^ cells), the large dot option, by FlowJo software, was used. FSC: forward scatter, SSC: side scatter. **Figure S3 **Representative images of two clinical cases. Panel A. Representative pictures of a clinical case of PB-MNCs therapy and right Chopart Amputation. From left to right: (i) before PB-MNCs therapy; (ii) at the time of the 1st treatment; (iii) at the 2nd treatment; (iv) at the 3rd treatment; (v) ulcer healing; (vi) definitive orthosis employing Below the Knee rigid casting with semi-rigid sole with high energy release to allow correct residual walking capacity. Panel B. Forefoot-mid foot gangrene in severe CLTI with no distal vessels run-off submitted to PB-MNC therapy. (i) Dorsal and (ii) plantar aspect of the foot; (iii) the aspect of the foot with secondary bone coverage and new dermal tissues coming from dermal substitute HA-based dressing. (iv) Dorsal and (v) plantar aspect of the foot at the indicated time after treatment. (vi) Correct orthesis for transmetatarsal amputation: custom-made shoes with lateral and medial reinforcement, biomechanics rigid rocker bottom sole, and fitting multilayer insole. **Figure S4** Probability of ulcer healing without a major amputation during the 1st year after treatment in the study population stratified by TcPO_2_ at the last treatment (panel A); during the whole follow-up period in the study population stratified by TcPO_2_ at the last treatment (panel B). Patients who died or drop out were not counted as “at-risk” (censored subjects). **Figure S5** Relative abundance of CD34^+^ subpopulations during PB-MNCs therapy stratified by ulcer healing without a major amputation after 1 year. Panel A. CD34^+^. Panel B. CD34^+^CXCR4^+^ HSPCs. The grey box represents patients with healing and the white box represents patients without healing at 1-year follow-up. No statistical differences were observed. **Table S1** Perfusion and cellular characterization at enrollement, stratifying study population according to ulcer healing without a major amputation at 1 year. **Table S2** Clinical outcomes occurred during follow-up, stratifying the study population according to TcPO_2_ levels after the last PB-MNCs treatment (< 40 mmHg; ≥ 40 mmHg).

## Data Availability

The datasets used and/or analyzed during the current study are available from the corresponding author on reasonable request.

## Abbreviations

ATK: Above-the-Knee
BM: Bone marrow
BTA: Below-the-Ankle
BTK: Below-the-Knee
CLTI: Critical limb-threatening ischemia
EDTA: Ethylene di-amine tetra-acetic acid
EVs: Extracellular vesicles
HA: Hyaluronic Acid
HSPCs: Hematopoietic stem and progenitor cells
PAD: Peripheral arterial disease
PB-MNCs: Peripheral blood mononuclear cells
PTA: Percutaneous Transluminal Angioplasty
RCT: Randomized controlled trial
RT: Room Temperature
SD: Standard deviation
TcPO_2_: Transcutaneous oxygen pressure
TUC: Texas University Class
