# Supplementary material for: Results of a prospective observational study of autologous peripheral blood mononuclear cell therapy for no-option critical limb-threatening ischemia and severe diabetic foot ulcers
Source: Cardiovasc Diabetol. 2022 Sep 28;21:196. doi: 10.1186/s12933-022-01629-y (PMC9516816; doi:10.1186/s12933-022-01629-y)
Supplement: Supplementary file 1 — Additional file 1: Figure S1 Panel A-B. Representative case of gangrene of the big toe. Panel C. PB-MNCs injection procedure was performed along the relevant axis below the knee, below the ankle, and closed to the wound area, at intervals of 1–2 cm and to a mean depth of 1.5–2 cm, using a 21G needle.Panel D. Patient submitted to big toe necrosectomy with bone exposure and dressing with Bioinductive Dermal Substitute based on Hyaluronic Acid. Figure S2 Representative dot plots for the gating strategy to detect the relative frequency (expressed as % of cells positive for the selected surface antigens and their combination) of the following PB-MNCs populations, as determined by multicolor flow cytometry. For rare events (CD34+ and CD34+CXCR4+ cells), the large dot option, by FlowJo software, was used. FSC: forward scatter, SSC: side scatter. Figure S3 Representative images of two clinical cases. Panel A. Representative pictures of a clinical case of PB-MNCs therapy and right Chopart Amputation. From left to right: (i) before PB-MNCs therapy; (ii) at the time of the 1st treatment; (iii) at the 2nd treatment; (iv) at the 3rd treatment; (v) ulcer healing; (vi) definitive orthosis employing Below the Knee rigid casting with semi-rigid sole with high energy release to allow correct residual walking capacity. Panel B. Forefoot-mid foot gangrene in severe CLTI with no distal vessels run-off submitted to PB-MNC therapy. (i) Dorsal and (ii) plantar aspect of the foot; (iii) the aspect of the foot with secondary bone coverage and new dermal tissues coming from dermal substitute HA-based dressing. (iv) Dorsal and (v) plantar aspect of the foot at the indicated time after treatment. (vi) Correct orthesis for transmetatarsal amputation: custom-made shoes with lateral and medial reinforcement, biomechanics rigid rocker bottom sole, and fitting multilayer insole. Figure S4 Probability of ulcer healing without a major amputation during the 1st year after treatment in the study populat [file 12933_2022_1629_MOESM1_ESM.docx]

**Online-Only Supplemental Material**

**Manuscript title:** Results of a prospective observational study of autologous peripheral blood mononuclear cell therapy for no-option critical limb-threatening ischemia and severe diabetic foot ulcers.

**List of the authors:** Andrea Panunzi, Fabiana Madotto, Elena Sangalli, Federica Riccio, Adriana Barbara Sganzaroli, Paolo Galenda, Amelia Bertulessi, Maria Francesca Barmina, Ornella Ludovico, Orazio Fortunato, Francesco Setacci, Flavio Airoldi, Davide Tavano, Laura Giurato, Marco Meloni, Luigi Uccioli, Antonino Bruno, Gaia Spinetti, and Carlo Maria Ferdinando Caravaggi.

**Supplemental Figures**

**Supplemental Figure 1**


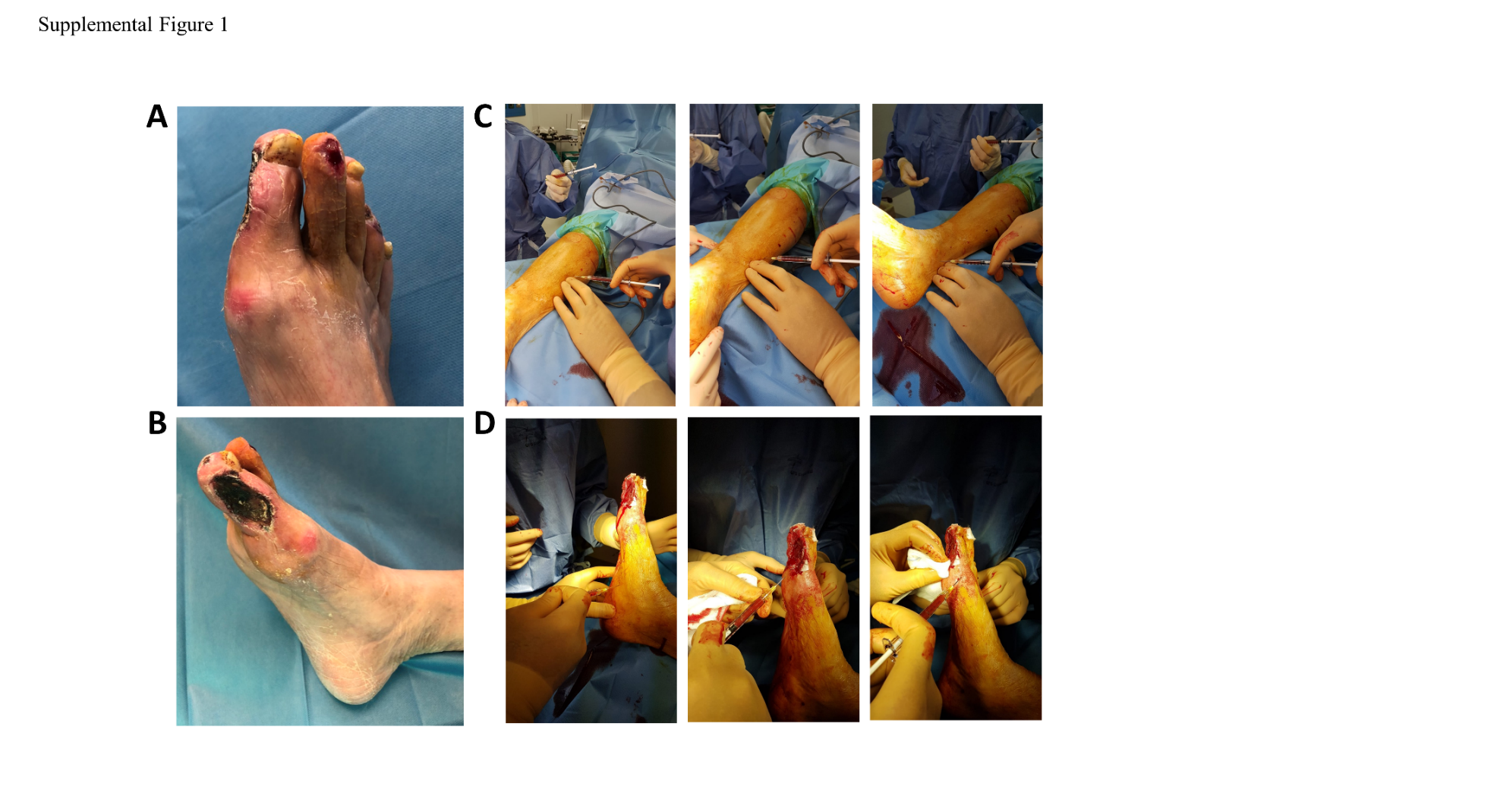


**Supplemental Figure 1.**

**Panel A-B.** Representative case of gangrene of the big toe.

**Panel C.** PB-MNCs injection procedure was performed along the relevant axis below the knee, below the ankle, and closed to the wound area, at intervals of 1–2 cm and to a mean depth of 1.5–2 cm, using a 21G needle.

**Panel D.** Patient submitted to big toe necrosectomy with bone exposure and dressing with Bioinductive Dermal Substitute based on Hyaluronic Acid.

**Supplemental Figure 2**


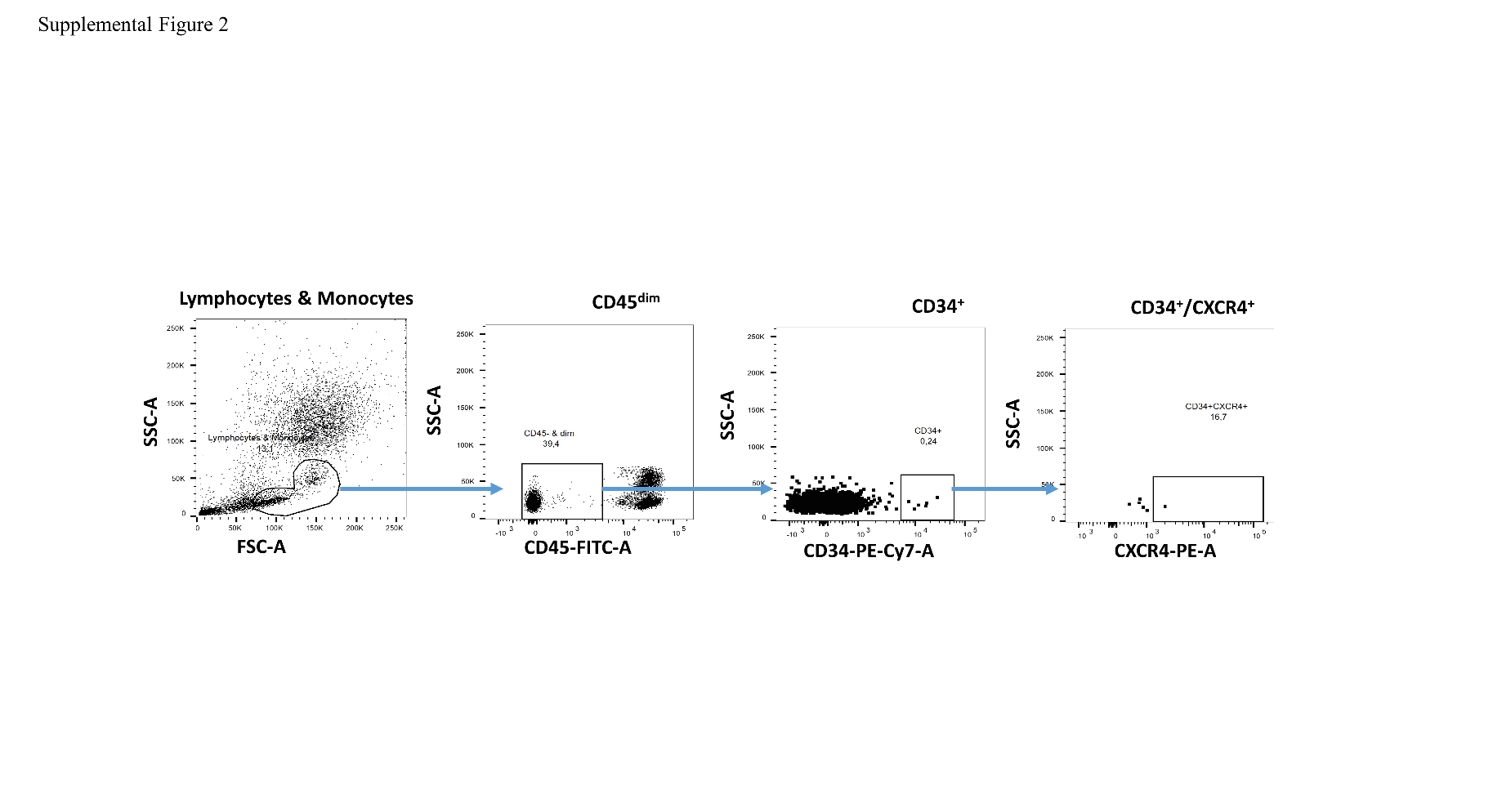


**Supplemental Figure 2.** Representative dot plots for the gating strategy to detect the relative frequency (expressed as % of cells positive for the selected surface antigens and their combination) of the following PB-MNCs populations, as determined by multicolor flow cytometry. For rare events (CD34^+^ and CD34^+^CXCR4^+^ cells), the large dot option, by FlowJo software, was used. FSC: forward scatter, SSC: side scatter.

**Supplemental Figure 3**


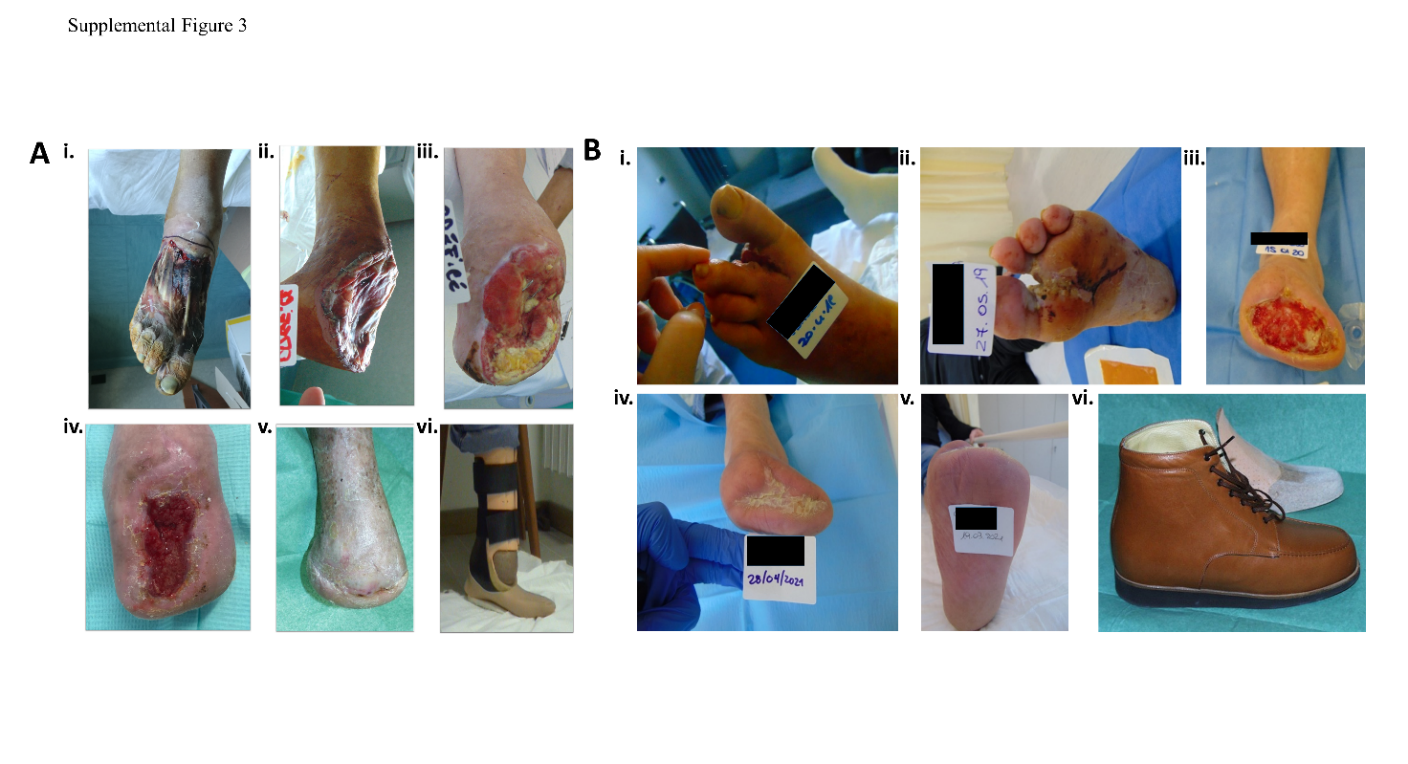


**Supplemental Figure 3.** Representative images of two clinical cases.

**Panel A.** Representative pictures of a clinical case of PB-MNCs therapy and right Chopart Amputation. From left to right: **i**) before PB-MNCs therapy; **ii**) at the time of the 1^st^ treatment; **iii**) at the 2^nd^ treatment; **iv**) at the 3^rd^ treatment; **v**) ulcer healing; **vi**) definitive orthosis employing Below the Knee rigid casting with semi-rigid sole with high energy release to allow correct residual walking capacity.

**Panel B**. Forefoot-mid foot gangrene in severe CLTI with no distal vessels run-off submitted to PB-MNC therapy. **i**) Dorsal and **ii**) plantar aspect of the foot; **iii**) the aspect of the foot with secondary bone coverage and new dermal tissues coming from dermal substitute HA-based dressing. **iv**) Dorsal and **v**) plantar aspect of the foot at the indicated time after treatment. **vi**) Correct orthesis for transmetatarsal amputation: custom-made shoes with lateral and medial reinforcement, biomechanics rigid rocker bottom sole, and fitting multilayer insole.

**Supplemental Figure 4**


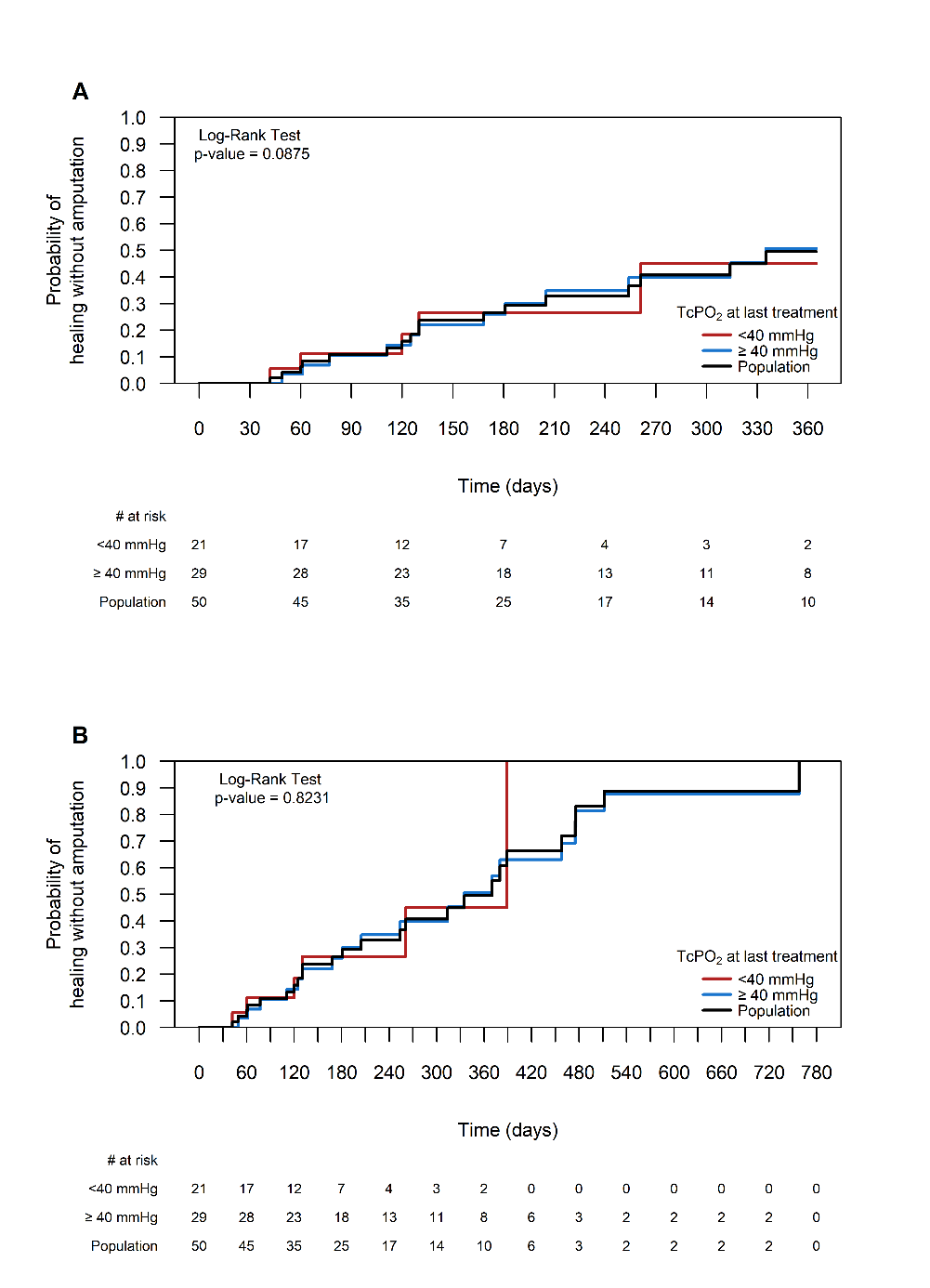


**Supplemental Figure 4.** Probability of ulcer healing without a major amputation during the first year after treatment in the study population stratified by TcPO_2_ at the last treatment (**panel A**); during the whole follow-up period in the study population stratified by TcPO_2_ at the last treatment (**panel B**). Patients who died or drop out were not counted as “at-risk” (censored subjects).

**Supplemental Figure 5**

**
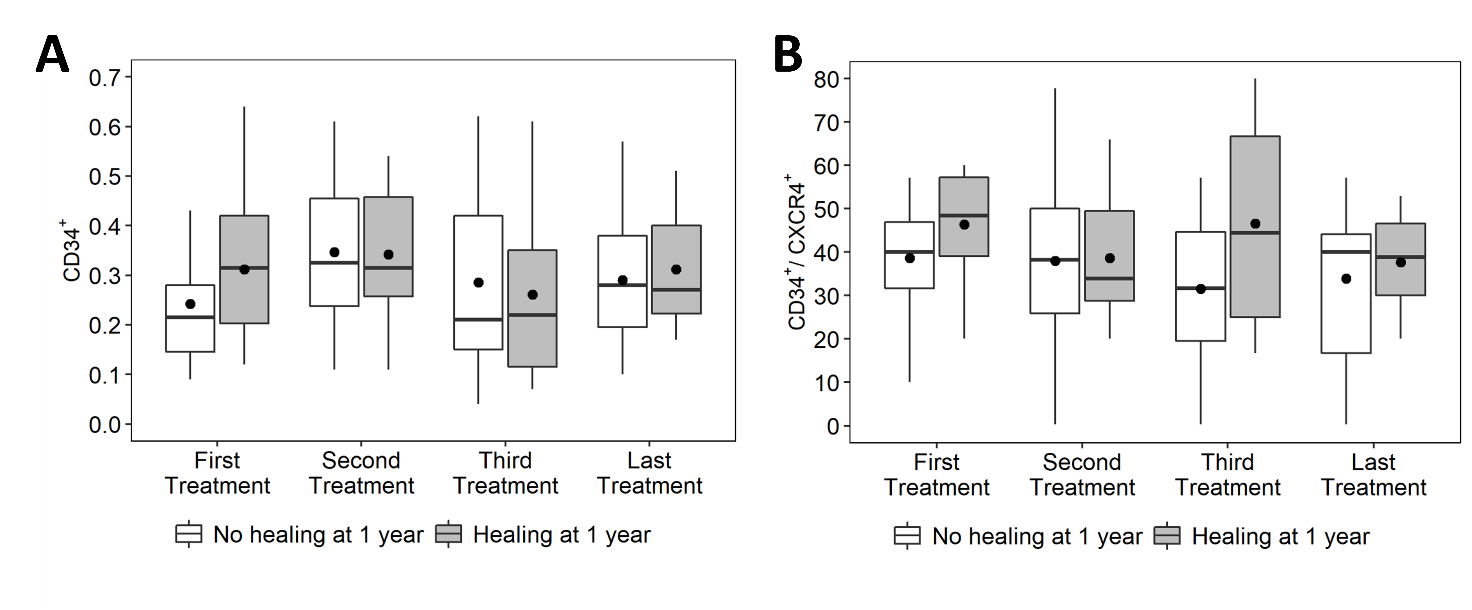
**

**Supplemental Figure 5.** Relative abundance of CD34^+^ subpopulations during PB-MNCs therapy stratified by ulcer healing without a major amputation after 1 year.

**Panel A.** CD34^+^.

**Panel B.** CD34^+^CXCR4^+^ HSPCs.

The grey box represents patients with healing and the white box represents patients without healing at 1-year follow-up. No statistical differences were observed.

**Supplemental Tables**

**Table S1.** Perfusion and cellular characterization at enrollement, stratifying study population according to ulcer healing without a major amputation at 1 year.

|  | **Healing without a major amputation at 1 year** | | **Study population** | **Comparison,**  **p-value** | |  |
| --- | --- | --- | --- | --- | --- | --- |
|  | **No** | **Yes** |  |  |  |  |
| N (%) | 33 (66.0) | 17 (34.0) | 50 (100.0) | - | |  |
| Age (years), mean ± SD | 74.67 ± 11.48 | 76.24 ± 6.48 | 75.20 ± 10.01 | 0.7974 | |  |
| Males, n (%) | 20 (60.6) | 11 (64.7) | 31 (62.0) | 0.7772 | |  |
| **PB-MNCs treatments, n (%)** | | | | | 0.7514 | |
| 1 | 9 (17.3) | 3 (17.6) | 12 (24.0) |  | |  |
| 2 | 7 (21.2) | 4 (23.5) | 11 (22.0) |  | |  |
| 3 | 17 (51.5) | 10 (58.8) | 27 (54.0) |  | |  |
| **TcPO_2_, mean ± SD** | | | | | | |
| At enrollment | 17.70 ± 12.15 | 16.24 ± 10.58 | 17.20 ± 11.55 | 0.8616 | |  |
| After the last PB-MNCs treatment | 34.61 ± 20.49 | 47.88 ± 22.05 | 39.12 ± 21.75 | **0.0395** | |  |
| **PB cell frequency*, mean ± SD** | | | | | | |
| CD34^+^ | 0.27 ± 0.19 | 0.31 ± 0.15 | 0.28 ± 0.18 | 0.1410 | |  |
| CD34^+^CXCR4^+^ | 40.80 ± 16.66 | 46.40 ± 12.71 | 42.48 ± 15.64 | 0.1070 | |  |
| **EVs**^‡^**, mean ± SD** |  |  |  |  | |  |
| EV size (nm) | 164.28 ± 46.48 | 150.16 ± 31.61 | 157.86 ± 10.15 | 0.4247 | |  |
| Total EV concentration (ml×10^10^) | 3.32 ± 1.58 | 3.88 ± 1.54 | 3.57 ± 1.55 | 0.3390 | |  |
| Small EV (30-100nM) (%) | 0.20 ± 0.22 | 0.25 ± 0.18 | 0.22 ± 0.20 | 0.6209 | |  |

*Abbreviations. EVs: extracellular vesicles; IQR interquartile range [1^st^ quartile-3^rd^ quartile]; PB: peripheral blood; PB-MNCs: peripheral blood mononuclear cells; SD: standard deviation.*

*** *CD34^+^ was not available for 5 patients (10%) s, CD34^+^CXCR4^+^ for 10 patients (20%). ‡ Exosomes evaluation was performed on 22 patients (44%).*

**Table S2.** Clinical outcomes occurred during follow-up, stratifying the study population according to TcPO_2_ levels after the last PB-MNCs treatment (< 40 mmHg; ≥ 40 mmHg).

|  | **TcPO_2_ levels** | | **Comparison,**  **p-value** |
| --- | --- | --- | --- |
|  | **< 40 mmHg** | **≥ 40 mmHg** |  |
| **At 1 year** | | | |
| Dead, n (%) | 8 (38.10) | 8 (27.59) | 0.4317 |
| Major amputation, n (%) |  |  |  |
| All patients (n=50) | 6 (28.57) | 2 (6.90) | 0.0557 |
| Alive at 1 year (n=34) | 6 (46.15) | 1 (4.76) | **0.0070** |
| Ulcer healing, n (%) |  |  |  |
| All patients (n=50) | 10 (47.62) | 12 (41.38) | 0.6609 |
| Alive at 1 year (n=34) | 10 (76.92) | 11 (52.38) | 0.2763 |
| Ulcer healing without a major amputation, n (%) |  |  |  |
| All patients (n=50) | 5 (23.81) | 12 (41.38) | 0.1955 |
| Alive at 1 year (n=34) | 5 (38.46) | 11 (52.38) | 0.4294 |
| Estimated* time to healing without a major amputation (days), mean ± SE | 219 ± 30 | 287 ± 20 | 0.0875 |
| **At the end of the follow-up** | | | |
| Dead, n (%) | 14 (66.67) | 10 (34.48) | **0.0246** |
| Major amputation, n (%) |  |  |  |
| All patients (n=50) | 7 (33.33) | 2 (6.90) | **0.0253** |
| Alive at the end of follow-up (n=26) | 4 (57.14) | 1 (5.26) | **0.0104** |
| Ulcer healing, n (%) |  |  |  |
| All patients (n=50) | 11 (52.38) | 19 (65.52) | 0.3494 |
| Alive at the end of follow-up (n=26) | 7 (100.00) | 16 (84.21) | 0.5396 |
| Ulcer healing without a major amputation, n (%) |  |  |  |
| All patients (n=50) | 6 (28.57) | 19 (65.52) | **0.0099** |
| Alive at the end of follow-up (n=26) | 3 (42.86) | 16 (84.21) | 0.0571 |
| Estimated* time to healing without a major amputation (days), mean ± SE | 287 ± 40 | 350 ± 46 | 0.8231 |

*Abbreviations. SD standard deviation; SE: standard error.*

** Time was estimated with Kaplan-Meier approach, considering deaths and drop-out as censored.*
